# Matrix convexity and unitary power dilations of Toeplitz-contractive operator tuples

**DOI:** 10.1007/s44146-024-00170-6

**Published:** 2024-11-29

**Authors:** Douglas Farenick

**Affiliations:** Department of Mathematics and Statistics, University of Regina, Regina, Saskatchewan S4S 0A2 Canada

**Keywords:** 47A20, 47A13, 46L07

## Abstract

Using works of T. Ando and L. Gurvits, the well-known theorem of P.R. Halmos concerning the existence of unitary dilations for contractive linear operators acting on Hilbert spaces is recast as a result for *d*-tuples of contractive Hilbert space operators satisfying a certain matrix-positivity condition. Such operator *d*-tuples satisfying this matrix-positivity condition are called, herein, Toeplitz-contractive, and a characterisation of the Toeplitz-contractivity condition is presented. The matrix-positivity condition leads to definitions of new distance-measures in several variable operator theory, generalising the notions of norm, numerical radius, and spectral radius to *d*-tuples of operators (commuting, for the spectral radius) in what appears to be a novel, asymmetric way. Toeplitz contractive operators form a noncommutative convex set, and a scaling constant $c_d$ for inclusions of the minimal and maximal matrix convex sets determined by a stretching of the unit circle $S^1$ across *d* complex dimensions is shown to exist.

## Introduction

The influential, foundational paper of Paul Halmos [17] in 1950 examined the notions of dilation, compression, and extension in the algebra ${\mathcal {B}}({\mathcal {H}})$ of bounded linear operators acting on a complex Hilbert space ${\mathcal {H}}$. If $T:{\mathcal {H}}\rightarrow {\mathcal {H}}$ is a bounded linear operator, then a *dilation* of *T* is a bounded linear operator $S:{\mathcal {K}}\rightarrow {\mathcal {K}}$ acting on a Hilbert space ${\mathcal {K}}$ that contains ${\mathcal {H}}$ as a subspace such that, for every $\xi \in H$,

1.1 $$\begin{aligned} T\xi =P_{{\mathcal {H}}} S\xi , \end{aligned}$$

where $P_{{\mathcal {H}}}$ is the linear selfadjoint projection operator on ${\mathcal {K}}$ whose range is ${\mathcal {H}}$ and kernel is ${\mathcal {H}}^\bot$. Conversely, given a bounded linear operator *S* acting on ${\mathcal {K}}$ and a subspace ${\mathcal {H}}$ of ${\mathcal {K}}$, equation (1.1) defines a bounded linear operator *T* on ${\mathcal {H}}$, which is called the *compression* of *S*. A dilation *S* of *T* is an *extension* of *T* if the subspace ${\mathcal {H}}$ of ${\mathcal {K}}$ is invariant under the action of *S*.

The general goal of dilation theory is to study an operator *T*, which may be difficult to analyse directly, by dilating *T* to an operator *S* that enjoys well understood properties, and then using *S* to analyse *T*. For example, if *S* is a dilation of *T*, then equation (1.1) indicates $\Vert T\Vert \le \Vert S\Vert$; thus, if *S* is a unitary operator, then every compression *T* of *S* is a contraction (i.e., $\Vert T\Vert \le 1$). The theorem below of Halmos [17] shows the converse is true.

### Theorem 1.1

(Halmos). If *T* is a contraction, then *T* has a unitary dilation.

Halmos’ proof of Theorem 1.1 is constructive in that he shows the dilating Hilbert space can be taken to be ${\mathcal {K}}={\mathcal {H}}\oplus {\mathcal {H}}$ and gives an explicit description of the unitary dilation *U* as a $2\times 2$ matrix of operators.

Because $2\times 2$ matrices of operators can be used to recover the norm of a single operator via the formula

1.2 $$\begin{aligned} \Vert T\Vert = \inf \left\{ \alpha \in {\mathbb {R}}_+\,|\,\left[ \begin{array}{cc} \alpha 1_{\mathcal {H}}&  T^* \\ T &  \alpha 1_{\mathcal {H}}\end{array}\right] \hbox { is a positive operator on } {\mathcal {H}}\oplus {\mathcal {H}}\right\} ,\nonumber \\ \end{aligned}$$

where $1_{\mathcal {H}}$ denotes the identity operator on ${\mathcal {H}}$, an equivalent formulation of Halmos’ Theorem can be made in terms of matrix positivity.

### Theorem 1.2

(Halmos, Alternative Version). The following statements are equivalent for a bounded linear operator *T* acting on a Hilbert space ${\mathcal {H}}$.

1. the operator matrix $$\left[ \begin{array}{cc} 1_{\mathcal {H}}&  T^* \\ T &  1_{\mathcal {H}}\end{array}\right] $$, acting on ${\mathcal {K}}={\mathcal {H}}\oplus {\mathcal {K}}$, is a positive operator on ${\mathcal {K}}$;
2. there exists a unitary operator $U\in {\mathcal {B}}({\mathcal {K}})$ such that *U* is a dilation of *T*.

The purpose of this paper is to bring together a variety of known results to show that a “matrix positivity” property leads to a version of Halmos’s Theorem 1.2 for not just one contraction, but for *d* contractive operators, leading to new distance measures involving operator *d*-tuples and new forms of joint numerical and spectral radii that mimic properties well-known to hold for single operators.

### Definition 1.3

(Toeplitz-contractive *d*-tuples). A *d*-tuple $\textsf{T}=(T_1,\dots T_d)$ of bounded linear operators on a Hilbert space ${\mathcal {H}}$ is *Toeplitz-contractive* if the Toeplitz operator-matrix

$$\begin{aligned} {\mathcal {T}}= \left[ \begin{array}{ccccc} 1_{\mathcal {H}}& \quad T_1^* & \quad T_2^* &  \quad \dots &  \quad T_d^* \\ T_1 & \quad 1_{\mathcal {H}}&  \quad T_1^* &  \quad \ddots & \quad \vdots \\ T_2 & \quad \ddots & \quad \ddots & \quad \ddots & \quad T_2^* \\ \vdots & \quad \ddots & \quad \ddots & \quad \ddots & \quad T_1^* \\ T_d & \quad \dots & \quad T_2 & \quad T_1 & \quad 1_{\mathcal {H}}\end{array}\right] \end{aligned}$$

is a positive operator on ${\mathcal {K}}=\displaystyle \bigoplus _1^{d+1}{\mathcal {H}}$.

Definition 1.3 is not the usual way one defines contractivity of an operator *d*-tuple. One of the most widely used definitions is that of a row contraction.

### Definition 1.4

(Row-contractive *d*-tuples). A *d*-tuple $\textsf{T}=(T_1,\dots T_d)$ of operators $T_k$ on ${\mathcal {H}}$ is *row contractive* if the linear map $\displaystyle \bigoplus _1^d{\mathcal {H}}\rightarrow {\mathcal {H}}$ given by

$$\begin{aligned} (\xi _1,\dots \,\xi _d) \longmapsto \sum _{k=1}^d T_k\xi _k \end{aligned}$$

is a contraction.

As with single operators, the row-contractivity condition has an equivalent formulation as a positivity condition: a *d*-tuple $\textsf{T}$ is row-contractive if and only if $1_{\mathcal {H}}-\sum \nolimits _{k=1}^d T_kT_k^*$ is a positive operator.

The simplest example of a Toeplitz-contractive *d*-tuple is the following one.

### Example 1.5

If $T\in {\mathcal {B}}({\mathcal {H}})$ is a contraction, then $\textsf{T}=(T, T^2, \dots , T^d)$ is Toeplitz-contractive.

### Proof

See [22, pp. 13–14] for a direct calculation showing $\textsf{T}$ is Toeplitz contractive. $\square$

### Corollary 1.6

If $\phi :{\mathcal {B}}({\mathcal {H}})\rightarrow {\mathcal {B}}({\mathcal {K}})$ is a unital completely positive linear map, and if $U\in {\mathcal {B}}({\mathcal {H}})$ is unitary, then

$$\begin{aligned} \phi (\textsf{U})=\left( \phi (U),\phi (U^2),\dots ,\phi (U^d)\right) \end{aligned}$$

is Toeplitz contractive, for every $d\in {\mathbb {N}}$.

### Proof

Because $\phi$ is completely positive, applying $\phi$ to each entry of the positive operator-matrix

$$\begin{aligned} {\mathcal {U}} = \left[ \begin{array}{ccccc} 1_{\mathcal {H}}& \quad U^* & \quad (U^2)^* & \quad \dots & \quad (U^d)^* \\ U & \quad 1_{\mathcal {H}}& \quad U ^* & \quad \ddots &  \quad \vdots \\ U^2 & \quad \ddots & \quad \ddots &  \quad \ddots & \quad (U^2)^* \\ \vdots & \quad \ddots &  \quad \ddots & \quad \ddots & \quad U ^* \\ U^d & \quad \dots & \quad U^2 & \quad U & \quad 1_{\mathcal {H}}\end{array}\right] \end{aligned}$$

yields a new positive Topeplitz matrix of operators, which is precisely the one induced by the *d*-tuple $\phi (\textsf{U})=\left( \phi (U),\phi (U^2),\dots ,\phi (U^d)\right)$. $\square$

Corollary 1.6 demonstrates that, for a given unitary $U\in {\mathcal {B}}({\mathcal {H}})$ and subspace ${\mathcal {L}}$ of ${\mathcal {H}}$, the compressions of $U^k$ to ${\mathcal {L}}$ lead to a sequence of operators $T_k=P_{{\mathcal {L}}}U^k_{\vert {\mathcal {L}}}$ on ${\mathcal {L}}$ such that the *d*-tuple $(T_1,\dots ,T_d)$ is Toeplitz contractive, for every $d\in {\mathbb {N}}$. The main result of this paper, Theorem 3.1, establishes the converse: that all Toeplitz-contractive *d*-tuples result through compressing powers of unitaries to a common subspace.

The study of norms and dilations of *d*-tuples of operators has a long history. In some works, such as [4], normal dilations are invoked to define a norm on operator *d*-tuples. This is also done in the present paper, resulting in a metric rather than a norm. Although a substantial literature has been developed concerning models for row-contractive *d*-tuples, dating back to [6], modern approaches explore, as in [9, 21], the close connection between normal dilations of *d*-tuples of operators and matrix convex sets, yielding applications to the matrix ranges [24] of operators.

## Further examples, notation, and definitions

The Cartesian product of *d*-copies of ${\mathcal {B}}({\mathcal {H}})$ is denoted by ${\mathcal {B}}({\mathcal {H}})^d$.

If $\phi :{\mathcal {B}}({\mathcal {H}})\rightarrow {\mathcal {B}}({\mathcal {K}})$ is a linear map and if $p\in {\mathbb {N}}$, then the *p*-th ampliation of $\phi$ is denoted by $\phi ^{[p]}$ and designates the linear map ${\mathcal {M}}_p\left( {\mathcal {B}}({\mathcal {H}})\right) \rightarrow {\mathcal {M}}_p\left( {\mathcal {B}}({\mathcal {K}}) \right)$ in which

$$\begin{aligned} \phi ^{[p]}\left( \left[ X_{ij}\right] _{i,j=1}^p\right) =\left[ \phi (X_{ij}) \right] _{i,j=1}^p, \end{aligned}$$

for every $p\times p$ operator matrix $\left[ X_{ij}\right] _{i,j=1}^p\in {\mathcal {M}}_p\left( {\mathcal {B}}({\mathcal {H}})\right)$. The map $\phi :{\mathcal {B}}({\mathcal {H}})\rightarrow {\mathcal {B}}({\mathcal {K}})$ is unital and completely positive (ucp) if $\phi (1_{\mathcal {H}})=1_{\mathcal {K}}$ and $\phi ^{[p]}$ is a positive linear map for every $p\in {\mathbb {N}}$.

Row-contractivity and Toeplitz-contractivity are defined in quite distinctive ways. For example, row-contractivity does not depend on the order of the operators in the *d*-tuple, whereas Toeplitz-contractivity does, as $T_1$ appears as an entry in the Toeplitz matrix far more frequently then $T_d$ does. The example below confirms these are indeed different concepts.

### Example 2.1

The notions of row-contractive and Toeplitz-contractive are distinct in that neither one of them implies the other.

### Proof

If *U* is a unitary operator, then Example 1.5 shows $(U, U^2)$ is Toeplitz-contractive. However, $UU^*+U^2(U^2)^*=2\cdot 1_{\mathcal {H}}$, which is not bounded above by $1_{\mathcal {H}}$. Hence, $(U, U^2)$ is Toeplitz-contractive, but not row-contractive.

Conversely, if *P* and *Q* are commuting nonzero projections such that $PQ=0$, then (*P*, *Q*) is row contractive. Consider the Toeplitz matrix

$$\begin{aligned} {\mathcal {P}} =\left[ \begin{array}{ccc} 1_{\mathcal {H}}& \quad P &  \quad Q \\ P &  \quad 1_{\mathcal {H}}& \quad P \\ Q & \quad P & \quad 1_{\mathcal {H}}\end{array}\right] . \end{aligned}$$

Select a unit vector $\xi \in {\mathcal {H}}$ in the range of *P*, and consider the state $\phi$ given by $\phi (X)=\langle X\xi ,\xi \rangle$, for bounded linear operators *X* on ${\mathcal {H}}$. Thus, $\phi (1_{\mathcal {H}})=\phi (P)=1$ and $\phi (Q)=0$; therefore, applying $\phi$ to each entry of ${\mathcal {P}}$ yields the $3\times 3$ complex matrix

$$\begin{aligned} \phi ^{[3]}({\mathcal {P}})= \left[ \begin{array}{ccc} 1 & \quad 1 & \quad 0 \\ 1& \quad 1& \quad 1 \\ 0& \quad 1& \quad 1 \end{array}\right] . \end{aligned}$$

However, the matrix $\phi ^{[3]}({\mathcal {P}})$ is not a positive operator on ${\mathbb {C}}^3$, which implies the matrix ${\mathcal {P}}$ cannot be a positive operator on ${\mathcal {H}}$ (because $\phi ^{[3]}$ is a positive linear map). Hence, the row contractive pair (*P*, *Q*) is not Toeplitz contractive. $\square$

By a similar argument, we note:

### Example 2.2

If $V_1$ and $V_2$ are isometries such that $V_1V_1^*+V_2V_2^*=1_{\mathcal {H}}$, then $(V_1,V_2)$ is row contractive but not Toeplitz contractive.

### Example 2.3

The following statements are equivalent for $T\in {\mathcal {B}}({\mathcal {H}})$:

1. (*T*, 0) is Toeplitz contractive;
2. $(T,T^*)$ is row contractive.

### Proof

The matrix

$$\begin{aligned} \left[ \begin{array}{ccc} 1_{\mathcal {H}}& \quad T^* & \quad 0 \\ T & \quad 1_{\mathcal {H}}& \quad T^* \\ 0 & \quad T & \quad 1_{\mathcal {H}}\end{array}\right] \end{aligned}$$

is positive if and only if $1_H-TT^*-T^*T$ is positive [12, Lemma 6.3]. $\square$

The operator-theoretic notions of normality, subnormality, and unitary take the following forms in several variables.

### Definition 2.4

A *d*-tuple $$\textsf{T}=(T_1,\dots T_d)\in {\mathcal {B}}({\mathcal {H}})^d$$ is:

1. *normal*, if $T_1$,..., $T_d$ are commuting normal operators;
2. *unitary*, if $T_1$,..., $T_d$ are commuting unitary operators;
3. *power unitary*, if there is a unitary operator $U\in {\mathcal {B}}({\mathcal {H}})$ such that $T_k=U^k$, for each $k=1,\dots , d$;
4. *subnormal*, if $T_1$,..., $T_d$ are pairwise commuting operators such that there exist a Hilbert space ${\mathcal {K}}$ containing ${\mathcal {H}}$ as a subspace and commuting normal operators $N_1,\dots ,N_d\in {\mathcal {B}}({\mathcal {K}})$ such that, for every $k=1,\dots , d$, ${\mathcal {H}}$ is an invariant subspace for $N_k$ and the restriction of $T_N$ to ${\mathcal {H}}$ is given by $T_k$.

It is known that a *d*-tuple of commuting subnormal operators may fail to be subnormal in the sense of (4) above; that is, there exist *d* commuting subnormal operators that fail to have an extension to *d* commuting normal operators.

Of the various definitions of joint spectrum for commuting operators, the following two are required. Recall from [26] that every *d*-tuple $\textsf{T}\in {\mathcal {B}}({\mathcal {H}})^d$ of pairwise commuting operators and $\lambda \in {\mathbb {C}}^d$ determine a chain complex, denoted by $E(\textsf{T}-\lambda ,{\mathcal {H}})$, called the Koszul complex.

### Definition 2.5

The *Gelfand joint spectrum* of a normal *d*-tuple $\textsf{N}=(N_1,\dots ,N_d)$ is the subset $\textrm{Sp}_G(\textsf{N})\subset \mathbb C^d$ of all $\lambda \in {\mathbb {C}}^d$ for which there exists a unital homomorphism $\pi :\textrm{C}^*(\textsf{N})\rightarrow {\mathbb {C}}$ such that $\lambda _j=\pi (N_j)$ for all $j=1,\dots ,d$, where $\textrm{C}^*(\textsf{N})$ is the unital abelian $\hbox {C}^*$-algebra generated by $N_1,\dots ,N_d$.

### Definition 2.6

The *Taylor joint spectrum* of a *d*-tuple $\textsf{T}=(T_1,\dots ,T_d)$ of pairwise commuting (possibly nonnormal) operators is the subset $\textrm{Sp}_T(\textsf{T})\subset {\mathbb {C}}^d$ of all $\lambda \in {\mathbb {C}}^d$ for which the Koszul complex $E(\textsf{T}-\lambda ,{\mathcal {H}})$ is not exact.

Lastly, the following forms of (joint) numerical range and matrix range [9, 24] are considered.

### Definition 2.7

Assume that $\textsf{T}=(T_1,\dots ,T_d)\in {\mathcal {B}}({\mathcal {H}})^d$. The *spatial numerical range* of $\textsf{T}$ is the set

2.1 $$\begin{aligned} W(\textsf{T})=\left\{ \left( \langle T_1\xi ,\xi \rangle , \dots , \langle T_1\xi ,\xi \rangle \right) \,|\,\xi \in {\mathcal {H}}, \, \Vert \xi \Vert =1\right\} ; \end{aligned}$$

the *numerical range* of $\textsf{T}$ is the set

2.2 $$\begin{aligned} W_1(\textsf{T})=\left\{ \left( \phi (T_1), \dots , \phi (T_d)\right) \,|\,\phi \hbox { is a state on }{\mathcal {B}}({\mathcal {H}}) \right\} ; \end{aligned}$$

and the *n*-*th matix range* of $\textsf{T}$ is the set

2.3 $$\begin{aligned} W_n(\textsf{T})=\left\{ \left( \phi (T_1), \dots , \phi (T_d)\right) \,|\,\phi \hbox { is a ucp map }{\mathcal {B}}({\mathcal {H}})\rightarrow {\mathcal {M}}_n({\mathbb {C}}) \right\} . \end{aligned}$$

The numerical ranges defined in (2.1) above have been extensively studied in operator theory, especially in the cases where ${\mathcal {H}}$ has finite dimension. In operator and matrix theory, $W(\textsf{T})$ is usually called the *joint numerical range* of $\textsf{T}$.

### Definition 2.8

The *universal positive* $n\times n$ *Toeplitz matrix* is the Toeplitz-matrix-valued function $T_n:S^1\rightarrow {\mathcal {M}}_n({\mathbb {C}})$ given by

2.4 $$\begin{aligned} T_n(z)=\left[ \begin{array}{cccccc} 1 & \quad z^{-1} & \quad z^{-2} &  \quad \dots & \quad z^{-n+2}&  \quad z^{-n+1} \\ z & \quad 1 & \quad z^{-1} & \quad z^{-2}&  \quad \dots &  \quad z^{-n+2} \\ z^2 &  \quad z &  \quad 1 &  \quad z^{-1} & \quad \ddots &  \quad \vdots \\ \vdots &  \quad \ddots & \quad \ddots & \quad \ddots & \quad \ddots & \quad z^{-2} \\ z^{n-2} &  &  \quad \ddots & \quad \ddots & \quad \ddots & \quad z^{-1} \\ z^{n-1} & \quad z^{n-2} &  \quad \dots & \quad z^{2} & \quad z& \quad 1 \end{array} \right] . \end{aligned}$$

The matrix $T_n(z)$ defined in (2.4) admits a factorisation of the form $T_n(z)=\gamma _n(z)\gamma _n(z)^*$, where

$$\begin{aligned} \gamma _n(z)=\left[ \begin{array}{c}1 \\ z \\ z^2 \\ \vdots \\ z^{n-1} \end{array}\right] . \end{aligned}$$

By functional calculus, if *U* is any unitary, then the operator matrix $T_n(U)$ is positive, and the first column below the (1, 1)-entry is a power-unitary operator $(n-1)$-tuple.

### Theorem 2.9

([7, Proposition 4.8(1)]). The extremal rays of the cone of positive $n\times n$ Toeplitz matrices are precisely the positive scalar multiples of Toeplitz matrices of the form $T_n(\lambda )$, for $\lambda \in S^1$.

To complete this section, let $S^1$ denote the unit circle in the complex plane and denote the multiplication operator $f\mapsto \varphi \cdot f$ on $L^2(S^1)$, where $\varphi :S^1\rightarrow \mathbb C$ is a continuous function, by $M_\varphi$. For each $k\in \mathbb Z$, let $\chi _k(z)=z^k$, for $z\in S^1$; thus, $\chi _{-k}=\overline{\chi _k}$ and $\chi _k=\chi ^k$, for every $k\in {\mathbb {Z}}$. By the Stone-Weierstrass Theorem, the unital abelian $\hbox {C}^*$-algebra $C(S^1)$ of complex continous functions $S^1\rightarrow {\mathbb {C}}$ coincides with the unital $\hbox {C}^*$-algebra $\textrm{C}^*(\chi )$ generated by $\chi$, which in turn is the group $\hbox {C}^*$-algebra generated by ${\mathbb {Z}}$. Thus, $\chi$ is a universal unitary, which is to say that if *U* is any unitary operator acting on ${\mathcal {B}}({\mathcal {H}})$, then there is a unital $*$-homomorphism $\pi :C(S^1)\rightarrow {\mathcal {B}}({\mathcal {H}})$ such that $\pi (\chi )^k=U^k$, for every $k\in {\mathbb {Z}}$. Because the unital $*$-homomorphism $\pi :C(S^1)\rightarrow {\mathcal {B}}\left( L^2(S^1)\right)$ in which $\pi (\varphi )=M_\varphi$, for every $\varphi \in C(S^1)$, is an isometry, the unitary operator $M_\chi$ is also a universal unitary. In representing $M_\chi$ as a doubly infinite (Laurent) matrix relative to the canonical orthonormal basis of $L^2(S^1)$, the operator $M\chi$ is unitarily equivalent to the bilateral shift operator *W* acting on the Hilbert space $\ell ^2({\mathbb {Z}})$. Thus, the bilateral shift operator is a universal unitary operator.

## The dilation theorem

The following theorem connects various results in the literature to characterise Toeplitz-contractive *d*-tuples in terms of norm conditions and unitary power dilations. The key step, asserting the existence of the dilation, is achieved in three separate ways: by applying Ando’s theorem [1, 2] on truncated moment problems; by appealing to Gurvits’ theorem [15, 16] on the separability of positive block-Toeplitz matrices; or by invoking the theory of pure completely positive linear maps [13].

### Theorem 3.1

The following statements are equivalent for a *d*-tuple $\textsf{T}=(T_1,\dots T_d)$ of bounded linear operators acting on a Hilbert space ${\mathcal {H}}$:

- the *d*-tuple $\textsf{T}$ is Toeplitz-contractive;
- for all $m\times m$ complex matrices $A_0,A_1,\dots A_d$, and all positive integers *m*,
  $$\begin{aligned} \left\| 1_{\mathcal {H}}\otimes A_0 + \sum _{k=1}^d T_k\otimes A_k\right\| \le \max _{z\in {\mathbb {C}},\,|z|=1}\left\| A_0 + \sum _{k=1}^d z^kA_k\right\| ; \end{aligned}$$
- there exists a Hilbert space ${\mathcal {K}}$ containing ${\mathcal {H}}$ as a subspace and a unitary operator *U* on ${\mathcal {K}}$ such that $U^k$ is a dilation of $T_k$, for each $k=1,\dots ,d$.

If the Hilbert space ${\mathcal {H}}$ is separable, then there is an additional equivalent assertion:

(d) for every $\varepsilon >0$, there is an isometry $V_\varepsilon :{\mathcal {H}}\rightarrow \ell ^2({\mathbb {Z}})$ such that
  $$\begin{aligned} \Vert T_k-V_\varepsilon ^* W^k V_\varepsilon \Vert <\varepsilon , \end{aligned}$$
  for each $k=1,\dots ,d$, where *W* denotes the bilateral shift operator on $\ell ^2({\mathbb {Z}})$.

Moreover, if ${\mathcal {H}}$ has finite dimension and the equivalent conditions (a)-(d) hold, then there exists a dilating Hilbert space ${\mathcal {K}}$, in (c), such that ${\mathcal {K}}$ has finite dimension.

### Proof

As mentioned above, there are three distinct proofs of the implication $\hbox {(a)}\Rightarrow \hbox {(c)}$.

The first proof is achieved using Ando’s Theorem [1, 2] on truncated moment problems, which shows that each $T_k$ is the image of $z^k\in C(S^1)$ under a ucp map $\phi :C(S^1)\rightarrow {\mathcal {B}}({\mathcal {H}})$. By expressing $\phi$ in terms of its Stinespring decomposition $\phi =V^*\pi V$, for some representation $\pi :C(S^1)\rightarrow {\mathcal {B}}({\mathcal {H}}_\pi )$ and linear isometry $V:{\mathcal {H}}\rightarrow {\mathcal {H}}_\pi$, then the dilation is achieved by taking ${\mathcal {K}}={\mathcal {H}}_\pi$, $U=\pi (z)$, $P=VV^*$, and considering *T* as the operator on the subspace $V({\mathcal {H}})\subset {\mathcal {K}}$ given by $\eta \mapsto VTV^*\eta$, for $\eta \in V({\mathcal {H}})$.

The second proof is via Gurvits’ proof of his theorem [15, 16] on separable positive block-Toeplitz matrices, under the hypothesis that ${\mathcal {H}}$ has finite dimension; in this case, Gurvits’ methods show the resulting dilating space ${\mathcal {K}}$ can be taken to have finite dimension.

The third proof is due to the author and McBurney [13], based on the theory of pure completely positive linear maps. The proof in [13] requires no restriction on the dimension of ${\mathcal {H}}$; however, if ${\mathcal {H}}$ has finite dimension, then it is shown in [13] that the dilating space ${\mathcal {K}}$ can also be taken to be finite-dimensional.

To prove the implication $\hbox {(c)}\Rightarrow \hbox {(b)}$, let $(U,\dots ,U^d)$ be a unitary dilation of $(T_1,\dots ,T_d)$. Thus, $T_k\xi = P_{\mathcal {H}}U^k\xi$, for every $\xi \in H$ and $k=1,\dots ,d$. If *m* is a positive integer and $A_0,A_1,\dots A_d$ are $m\times m$ complex matrices, then the operators

$$\begin{aligned} 1_{\mathcal {H}}\otimes A_0 + \sum _{k=1}^d T_k\otimes A_k \,\hbox { and }\, 1_{\mathcal {K}}\otimes A_0 + \sum _{k=1}^d U^k\otimes A_k \end{aligned}$$

act on the Hilbert spaces ${\mathcal {H}}\otimes {\mathbb {C}}^m$ and ${\mathcal {K}}\otimes {\mathbb {C}}^m$, respectively. Moreover,

$$\begin{aligned} 1_{\mathcal {H}}\otimes A_0 + \sum _{k=1}^d T_k\otimes A_k \,\hbox { is a compression of }\, 1_{\mathcal {K}}\otimes A_0 + \sum _{k=1}^d U^k\otimes A_k \end{aligned}$$

to the subspace ${\mathcal {H}}\otimes {\mathbb {C}}^m$ of ${\mathcal {K}}\otimes {\mathbb {C}}^m$. Hence,

$$\begin{aligned} \left\| 1_{\mathcal {H}}\otimes A_0 + \sum _{k=1}^d T_k\otimes A_k \right\| \le \left\| 1_{\mathcal {K}}\otimes A_0 + \sum _{k=1}^d U^k\otimes A_k \right\| . \end{aligned}$$

Because $\textsf{U}=(U,\dots , U^d)$ is a *d*-tuple of pairwise commuting unitary operators, the maximal ideal space ${\mathfrak {M}}$ of the abelian $\hbox {C}^*$-algebra $\textrm{C}^*(\textsf{U})$ is homeomorphic to $\hbox {Sp}_G(\textsf{U})$ under the map $\rho \mapsto (\rho (U),\dots ,\rho (U^d))$. Hence, every operator $U^k$ is a complex-valued continuous function on ${\mathfrak {M}}$ whereby $\rho \mapsto \rho (U^k)$. Because the unital $\hbox {C}^*$-algebra $C({\mathfrak {M}})\otimes {\mathcal {M}}_m({\mathbb {C}})$ is isometrically isomorphic to the $\hbox {C}^*$-algebra of continuous functions on ${\mathfrak {M}}$ with values in ${\mathcal {M}}_m({\mathbb {C}})$, we deduce that

$$\begin{aligned} \left\| 1_{\mathcal {K}}\otimes A_0 + \sum _{k=1}^d U^k\otimes A_k \right\| = \max _{\rho \in {\mathfrak {M}}} \left\| A_0+\sum _{k=1}^d \rho (U^k) A_k \right\| . \end{aligned}$$

Furthermore, because $\rho (U^k)=\rho (U)^k$ and

$$\begin{aligned} \hbox {Sp}_G(\textsf{U})=\left\{ (\lambda ,\lambda ^2,\dots ,\lambda ^d)\,|\,\lambda \in \hbox {Sp}_G(U)=\hbox {Sp}(U)\right\} \subseteq \left\{ (z,z^2,\dots ,z^d)\,|\,z \in S^1\right\} , \end{aligned}$$

we obtain

$$\begin{aligned} \left\| 1_{\mathcal {H}}\otimes A_0 + \sum _{k=1}^d T_k\otimes A_k\right\| \le \max _{z\in {\mathbb {C}},\,|z|=1}\left\| A_0 + \sum _{k=1}^d z^kA_k\right\| , \end{aligned}$$

thereby completing the proof of the implication $\hbox {(c)}\Rightarrow \hbox {(b)}$.

To prove the implication $\hbox {(b)}\Rightarrow \hbox {(a)}$, assume that $T_1,\dots ,T_d\in {\mathcal {B}}({\mathcal {H}})$ are such that inequality (b) holds for all matrices $A_k\in {\mathcal {M}}_m({\mathbb {C}})$ and positive integers *m*. Let ${\mathcal {Z}}_d$ be the finite-dimensional subspace of $C(S^1)$ spanned by the continuous functions $\chi _k(z)=z^k$, for $k=0,1,\dots , d$, for $z\in S^1$. By the linear independence of $\chi _0,\dots ,\chi _d$, there is a unital linear map $\psi :\mathcal Z_d\rightarrow {\mathcal {B}}({\mathcal {H}})$ in which $\phi (\chi _k)=T_k$, for $k=1,\dots , d$. Inequality (b) asserts that $\psi$ is completely contractive; hence, the map $\phi :{\mathcal {Z}}_d+{\mathcal {Z}}_d^*\rightarrow {\mathcal {B}}({\mathcal {H}})$ given by $\phi (f+g^*)=\psi (f)+\psi (g)^*$ is unital and completely positive, and has the property that $\phi (\chi _k)=T_k$ and $\phi (\chi _k^*)=T_k^*$, for $k=1,\dots , d$. Because $\chi _k=\chi _1^k$, for $k=1,\dots , d$, the tuple $(\chi _1,\dots ,\chi _d)$ of unitaries is Toeplitz contractive; hence, so is the *d*-tuple $(\phi (\chi _1),\dots ,\phi (\chi _d))=(T_1,\dots , T_d)$, by the complete positivity of the linear map $\phi$.

To complete the proof, assume that ${\mathcal {H}}$ is a separable Hilbert space. The $\hbox {C}^*$-algebra generated by the multiplication operator $M_\chi$ is $\{M_\varphi \,|\,\varphi \in C(S^1)\}$, which is a faithful $*$-representation of $C(S^1)$, and $\textrm{C}^*(M_\chi )$ contains no compact operators other than 0. Thus, for any unital completely positive linear map $\phi :\textrm{C}^*(M_\chi )\rightarrow {\mathcal {B}}({\mathcal {H}})$, there exists a sequence of isometric operators ${\tilde{V}}_n:{\mathcal {H}}\rightarrow L^2(S^1)$ such that

3.1 $$\begin{aligned} \lim _{n\rightarrow \infty }\Vert \phi (M_\varphi )-\tilde{V}_n^*M_\varphi {\tilde{V}}_n\Vert =0, \end{aligned}$$

for every $\varphi \in C(S^1)$ [8, Theorem II.5.3], [27]. In particular, assuming $\textsf{T}$ is Toeplitz contractive, the previous paragraph shows the existence of a unital completely positive linear map $\phi :\textrm{C}^*(M_\chi )\rightarrow {\mathcal {B}}({\mathcal {H}})$ such that $T_k=\phi (M_{\chi _k})$, for $k=1,\dots , d$. Hence, if $\tilde{U}:\ell ^2({\mathbb {Z}})\rightarrow L^2(S^1)$ is the unitary operator for which $W={\tilde{U}}^*M_\chi {\tilde{U}}$, where *W* is the bilateral shift, then (3.1) becomes

3.2 $$\begin{aligned} \lim _{n\rightarrow \infty }\Vert T_k-V_n^*W^kV_n\Vert =0, \end{aligned}$$

for $k=1,\dots ,d$, where each $V_n:{\mathcal {H}}\rightarrow \ell ^2({\mathbb {Z}})$ is the isometry given by $V_n={\tilde{U}}{\tilde{V}}_n$. $\square$

Items (b) and (d) in Theorem 3.1 above are natural outcomes of similar ideas in [11, Theorem 4.1] and [9, Theorem 5.2], respectively. The assertion about Toeplitz contractive *d*-tuples acting on a finite-dimensional Hilbert space having a unitary power dilation that also acts on a space of finite dimension can be proved entirely algebraically, as in [15], or by means of convex analysis, as in [13]. The latter approach is not unlike the recent work of Hartz and Lupini [19], where matrix convexity is shown to play a prominent role in the dilation theory for operators acting on finite-dimensional Hilbert spaces.

It is well known that the unitary dilation *U* of a contraction *T* explicitly constructed by Halmos in [17] does not have the property that $U^n$ dilates $T^n$, for every $n\in {\mathbb {N}}$. In this respect, assertion (c) of Theorem 3.1 may be viewed as “Halmos dilation theorem in several variables.”

### Corollary 3.2

If *T* is a contraction acting on a Hilbert space ${\mathcal {H}}$, then there exists a Hilbert space ${\mathcal {K}}$ containing ${\mathcal {H}}$ as a subspace and a unitary operator $U\in {\mathcal {B}}({\mathcal {K}})$ such that

3.3 $$\begin{aligned} T^k=P_{\mathcal {H}}U^k_{\vert {\mathcal {H}}}, \end{aligned}$$

for all $$k=1,\dots , d$$.

Furthermore, if ${\mathcal {H}}$ has finite dimension, then the dilating Hilbert space ${\mathcal {K}}$ can be chosen to have finite dimension.

### Proof

Example 1.5 shows the *d*-tuple $\textsf{T}=(T,T^2,\dots ,T^d)$ is Toeplitz contractive. Thus, by Theorem 3.1, $\textsf{T}$ has a dilation to a power unitary *d*-tuple $\textsf{U}=(U,\dots , U^d)$, and the dilating space ${\mathcal {K}}$ has finite dimension, if ${\mathcal {H}}$ has finite dimension. $\square$

Corollary 3.2 is a truncated version of the famous dilation theorem of Sz.-Nagy [25], which asserts every contraction *T* admits a unitary dilation *U* for which (3.3) holds for all $k\in {\mathbb {N}}$, not just for $k=1,\dots ,d$. The dilating Hilbert space ${\mathcal {K}}$ in Sz.-Nagy’s theorem is generally infinite-dimensional, even if ${\mathcal {H}}$ has finite dimension—for example, this is the case if *T* is a non-unitary contraction. Therefore, the interest in Corollary 3.2 lies in the proof, independent of Sz.-Nagy’s theorem and of the similar result of Egerváry [10], of the existence of a unitary power dilation, up to *d* powers, and the assertion that ${\mathcal {K}}$ can be chosen to have finite dimension, if ${\mathcal {H}}$ has finite dimension.

A similar truncated version of Berger’s “strange dilation theorem” [3] holds for numerical contractions on finite-dimensional spaces. However, the only new information imparted in Corollary 3.3 below is the assertion concerning the finite-dimensionality of the dilating space, if the original space has finite dimension. The existence of the dilation is not given by a new argument; in this regard, Corollary 3.3 is in the same vein as [9, Theorem 7.1], which shows that once a normal dilation is known to exist, a normal dilation to a finite-dimensional space can be found.

### Corollary 3.3

If *S* is an operator acting on a finite-dimensional Hilbert space ${\mathcal {H}}$ such that $w(S)\le 1$, then there exists a finite-dimensional Hilbert space ${\mathcal {K}}$ containing ${\mathcal {H}}$ as a subspace and a unitary operator $U\in {\mathcal {B}}({\mathcal {K}})$ such that

3.4 $$\begin{aligned} S^k=2P_{\mathcal {H}}U^k_{\vert {\mathcal {H}}}, \end{aligned}$$

for all $$k=1,\dots , d$$.

## The Toeplitz modulus and numerical radius on ${\mathcal {B}}({\mathcal {H}})^d$

### Definition 4.1

The *Toeplitz modulus* of $\textsf{T}=(T_1,\dots ,T_d)\in {\mathcal {B}}({\mathcal {H}})^d$ is the nonnegative real number $\rho (\textsf{T})$ defined by

4.1 $$\begin{aligned} \rho (\textsf{T})= \inf \left\{ \alpha \in {\mathbb {R}}_+\,|\, \left[ \begin{array}{ccccc} \alpha 1_{\mathcal {H}}& \quad T_1^* & \quad T_2^* &  \quad \dots & \quad T_d^* \\ T_1 & \quad \alpha 1_{\mathcal {H}}& \quad T_1^* & \quad \ddots &  \quad \vdots \\ T_2 &  \quad \ddots & \quad \ddots & \quad \ddots &  \quad T_2^* \\ \vdots &  \quad \ddots &  \quad \ddots & \quad \ddots & \quad T_1^* \\ T_d &  \quad \dots &  \quad T_2 &  \quad T_1 & \quad \alpha 1_{\mathcal {H}}\end{array}\right] \hbox { is positive}\right\} .\nonumber \\ \end{aligned}$$

In light of Definition 4.1, an operator *d*-tuple $\textsf{T}$ is Toeplitz contractive if and only if $\rho (\mathsf{T)}\le 1$.

Some basic properties of the Toeplitz modulus are presented in the next few results. To facilitate their proofs, the following notation shall be used.

For any $\textsf{T}=(T_1,\dots , T_d)\in {\mathcal {B}}({\mathcal {H}})^d$ and $\alpha \in \mathbb R_+$, denote ${\mathcal {T}}_\alpha$ by

4.2 $$\begin{aligned} {\mathcal {T}}_\alpha = \left[ \begin{array}{ccccc} \alpha 1_{\mathcal {H}}& \quad T_1^* &  \quad T_2^* &  \quad \dots & \quad T_d^* \\ T_1 &  \quad \alpha 1_{\mathcal {H}}&  \quad T_1^* &  \quad \ddots &  \quad \vdots \\ T_2 &  \quad \ddots & \quad \ddots & \quad \ddots &  \quad T_2^* \\ \vdots & \quad \ddots & \quad \ddots & \quad \ddots & \quad T_1^* \\ T_d &  \quad \dots & \quad T_2 & \quad T_1 & \quad \alpha 1_{\mathcal {H}}\end{array}\right] . \end{aligned}$$

Thus, the defining condition (4.1) for the Toeplitz-modulus of $\textsf{T}$ becomes

4.3 $$\begin{aligned} \rho (\textsf{T})= \inf \left\{ \alpha \in {\mathbb {R}}_+\,|\,\mathcal T_\alpha \hbox { is a positive operator}\right\} . \end{aligned}$$

### Proposition 4.2

Suppose $\textsf{T}=(T_1,\dots ,T_d)\in {\mathcal {B}}({\mathcal {H}})^d$ and define $\textsf{T}^*=(T_1^*,\dots ,T_d^*)$.

1. If $$d=1$$, then $$\rho (\textsf{T})=\Vert T_1\Vert $$.
2. $$\rho (\textsf{T}^*)=\rho (\textsf{T})$$.
3. If $1\le q\le d$ and $1\le i_1< \cdots <i_q\le d$, then
  $$\begin{aligned} \rho \left( (T_{i_1}, \dots , T_{i_q})\right) \le \rho (\textsf{T}), \end{aligned}$$
  and $\Vert T_k\Vert \le \rho (\textsf{T})$, for every $k=1,\dots , d$.
4. If $\phi :{\mathcal {B}}({\mathcal {H}})\rightarrow {\mathcal {B}}({\mathcal {K}})$ is a ucp map, the $\rho \left( \phi (\textsf{T}) \right) \le \rho (\textsf{T})$.
5. $\rho (\textsf{T})=0$ if and only if $\textsf{T}=0$.

### Proof

The first assertion was noted earlier in equation (1.2).

For (2), for each $\alpha \in {\mathbb {R}}_+$, the Toeplitz matrices of operators ${\mathcal {T}}_\alpha ^*$ and ${\mathcal {T}}_\alpha$ are unitarily equivalent (via the selfadjoint permutation of $\displaystyle \bigoplus _1^d{\mathcal {H}}$ that repositions the direct summands of $\displaystyle \bigoplus _1^d{\mathcal {H}}$ in reverse order). Hence, ${\mathcal {T}}_\alpha ^*$ is positive if and only if ${\mathcal {T}}_\alpha$ is positive.

To prove (3), assume $1\le i_1<i_2<\cdots < i_q\le d$ and let $\textsf{T}({\mathcal {I}})=(T_{i_1},T_{i_2},\dots ,T_{i_q})$. Thus, for each $\alpha \in {\mathbb {R}}_+$, the operator matrix ${\mathcal {T}}(\mathcal I)_\alpha$ is a $q\times q$ principal submatrix of $\mathcal T_\alpha$. Thus, the positivity of ${\mathcal {T}}_\alpha$ implies the positivity of ${\mathcal {T}}({\mathcal {I}})_\alpha$. Hence, for any $\alpha \in {\mathbb {R}}_+$ for which ${\mathcal {T}}_\alpha$ is positive, we have $\rho \left( \textsf{T}({{\mathcal {I}}})\right) \le \alpha$, and so $\rho \left( \textsf{T}({{\mathcal {I}}})\right) \le \rho (\textsf{T})$.

The proof (4) is similar to the proof of (3): if $\alpha \in \mathbb R_+$ is such that ${\mathcal {T}}_\alpha$ is positive, then $\Phi ({\mathcal {T}})_\alpha$ is also positive, where $\Phi (\mathcal T)_\alpha$ is the Toeplitz matrix of operators obtained by applying $\phi$ to each entry of ${\mathcal {T}}_\alpha$.

Select $k\in \{1,\dots ,d\}$. Using (3) with ${\mathcal {I}}$ determined by the singleton set $\{k\}$, we have $\Vert T_k\Vert \le \rho (\textsf{T})$. Hence, $\rho (\textsf{T})=0$ if and only if $\textsf{T}=0$, thereby proving (5). $\square$

An important, interesting property of the Toeplitz modulus is that it is a convex function ${\mathcal {B}}({\mathcal {H}})^d\rightarrow {\mathbb {R}}$.

### Proposition 4.3

If $\textsf{A},\textsf{S}, \textsf{T}\in {\mathcal {B}}({\mathcal {H}})^d$ and $a,s,t\in {\mathbb {R}}_+$, then

$$\begin{aligned} \rho (a\textsf{A})=a\rho (\textsf{A}) \hbox { and } \rho (s\textsf{S}+t\textsf{T}) \le s\rho (\textsf{S})+t\rho (\textsf{T}). \end{aligned}$$

### Proof

The equation $\rho (a\textsf{A})=a\rho (\textsf{A})$ holds trivially if $a=0$. If $a>0$, then

$$\begin{aligned} \left[ \begin{array}{ccccc} \alpha 1_{\mathcal {H}}&  aA_1^* &  aA_2^* &  \dots & a A_d^* \\ aA_1 &  \alpha 1_{\mathcal {H}}&  aA_1^* &  \ddots &  \vdots \\ aA_2 &  \ddots &  \ddots &  \ddots &  aA_2^* \\ \vdots &  \ddots &  \ddots &  \ddots &  aA_1^* \\ a A_d &  \dots &  aA_2 &  aA_1 &  \alpha 1_{\mathcal {H}}\end{array}\right] = a \left[ \begin{array}{ccccc} \frac{\alpha }{a} 1_{\mathcal {H}}&  A_1^* &  A_2^* &  \dots &  A_d^* \\ A_1 &  \frac{\alpha }{a} 1_{\mathcal {H}}&  A_1^* &  \ddots &  \vdots \\ A_2 &  \ddots &  \ddots &  \ddots &  A_2^* \\ \vdots &  \ddots &  \ddots &  \ddots &  A_1^* \\ A_d &  \dots &  A_2 &  A_1 &  \frac{\alpha }{a} 1_{\mathcal {H}}\end{array}\right] =a {\mathcal {A}}_{\frac{\alpha }{a}}. \end{aligned}$$

Because the matrix on the left is positive if and only if the matrix ${\mathcal {A}}_{\frac{\alpha }{a}}$ is positive, we have

$$\begin{aligned} \rho (a \textsf{A}) = a \cdot \inf \left\{ \frac{\alpha }{a}\,| \mathcal A_{\frac{\alpha }{a}} \hbox { is positive}\right\} = a\rho (\textsf{A}). \end{aligned}$$

To complete the proof, it is sufficient to show that $\rho$ is subadditive, as $\rho (a\textsf{A})=a\rho (\textsf{A})$ for every $a\in {\mathbb {R}}_+$. To this end, select $\textsf{S}, \textsf{T}\in {\mathcal {B}}({\mathcal {H}})^d$ and suppose $\beta ,\gamma \in {\mathbb {R}}_+$ are such that ${\mathcal {S}}_\beta$ and ${\mathcal {T}}_\gamma$ are positive operators. Thus, the operator ${\mathcal {S}}_\beta +{\mathcal {T}}_\gamma$ is also positive. Let $\alpha =\beta +\gamma$. Then by the defining condition (4.1),

$$\begin{aligned} \rho (\textsf{S}+\textsf{T}) \le \alpha = \beta +\gamma . \end{aligned}$$

As the inequality above is true for any $\beta ,\gamma \in {\mathbb {R}}_+$ for which ${\mathcal {S}}_\beta$ and ${\mathcal {T}}_\gamma$ are positive operators, then passing to the infima of all such $\beta$ and $\gamma$ leads to

$$\begin{aligned} \rho (\textsf{S}+\textsf{T}) \le \rho (\textsf{S})+\rho (\textsf{T}), \end{aligned}$$

which shows that $\rho$ is subadditive. $\square$

One drawback of the Toeplitz modulus is that it fails to be a norm, or even a metric, if $d>1$, as the following example shows.

### Example 4.4

(Asymmetry) If ${\mathcal {H}}={\mathbb {C}}^2$ and $$\sigma =\left[ \begin{array}{cc} 0& 1 \\ 1& 0 \end{array}\right] $$, then the pair $(\sigma , 1_2)$ is Toeplitz contractive, but $(-\sigma ,-1_2)$ is not. Moreover,

$$\begin{aligned} \rho \left( (\sigma , 1_2)\right) =1<\rho \left( -(\sigma , 1_2)\right) . \end{aligned}$$

### Proof

Because $\sigma$ is a selfadjoint unitary, the pair $(\sigma , 1_2)$ has the form $(U,U^2)$, which is Toeplitz-contractive. Hence,

$$\begin{aligned} \left[ \begin{array}{ccc} 1_2 & \quad \sigma & \quad 1_2 \\ \sigma & \quad 1_2 & \quad \sigma \\ 1_2 & \quad \sigma & \quad 1_2 \end{array}\right] \end{aligned}$$

is a positive operator. On the other hand, the block-Toeplitz matrix

$$\begin{aligned} \left[ \begin{array}{ccc} 1_2 & \quad -\sigma &  \quad -1_2 \\ -\sigma & \quad 1_2 & \quad -\sigma \\ -1_2 &  \quad -\sigma &  \quad 1_2 \end{array}\right] \end{aligned}$$

has an eigenvalue of $-1$. Thus, $(-\sigma ,-1_2)$ is not a Toeplitz contractive pair, implying its Toeplitz-modulus exceeds 1.

To show $\rho \left( (\sigma ,1_2)\right) =1$, write $\textsf{T}=(\sigma ,1_2)$ and suppose $\alpha \in {\mathbb {R}}_+$ is such that the matrix ${\mathcal {T}}_\alpha$ is positive. Let $\xi \in {\mathbb {C}}^2$ be a unit (eigen)vector satisfying $\sigma \xi =\xi$, and let $\phi$ denotes the vector state $X\mapsto \langle X\xi ,\xi \rangle$. Applying the ampliation $\phi ^{[3]}$ to the positive matrix $\mathcal T_\alpha$ yields the positive matrix

$$\begin{aligned} \left[ \begin{array}{ccc} \alpha & \quad 1 &  \quad 1 \\ 1 & \quad \alpha & \quad 1 \\ 1 & \quad 1 &  \quad \alpha \end{array}\right] . \end{aligned}$$

However, by considering the upper-left $2\times 2$ submatrix, the matrix above is positive only if $\alpha \ge 1$. Hence, $1\le \rho \left( (\sigma ,1_2)\right) \le 1$. $\square$

Thus, if one were to use the Toeplitz-modulus as a distance measure, it may happen that the distance from $\textsf{S}$ to $\textsf{T}$ is different from the distance from $\textsf{T}$ to $\textsf{S}$. A remedy to this lack of symmetry in the set of Toeplitz-contractive operators is to take an average.

### Proposition 4.5

The formula $D^{\rho }(\textsf{S},\textsf{T})=\frac{1}{2}\left( \rho (\textsf{S}-\textsf{T})+\rho (\textsf{T}-\textsf{S}) \right)$ defines a metric on ${\mathcal {B}}({\mathcal {H}})^d$.

### Proof

The only non-obvious property of a metric to verify is the triangle inequality. If $\textsf{S}, { T}, \textsf{R}\in {\mathcal {B}}({\mathcal {H}})^d$, then

$$\begin{aligned} \begin{array}{rcl} D^\rho (\textsf{S}, \textsf{T}) & =&  \frac{1}{2}\rho (\textsf{S}- { T}) + \frac{1}{2}\rho (\textsf{T}- \textsf{S}) \\   & & \\ & =&  \frac{1}{2}\left( \rho ([\textsf{S}- \textsf{R}]+[\textsf{R}-\textsf{T}]) + \rho ([\textsf{T}- \textsf{R}]+[\textsf{R}-\textsf{S}]) \right) \\   & &  \\ & \le &  \frac{1}{2}\left( \rho ([\textsf{S}- \textsf{R}])+\rho ([\textsf{R}-\textsf{T}]) + \rho ([\textsf{T}- \textsf{R}])+\rho ([\textsf{R}-\textsf{S}]) \right) \\   & &  \\ & =&  \frac{1}{2}\left( \rho ([\textsf{S}- \textsf{R}])+\rho ([\textsf{R}-\textsf{S}]\right) + \frac{1}{2}\left( \rho ([\textsf{T}- \textsf{R}])+\rho ([\textsf{R}-\textsf{T}])\right) \\   & &  \\ & =&  D^\rho (\textsf{S}, \textsf{R}) + D^\rho (\textsf{R}, \textsf{T}), \end{array} \end{aligned}$$

where the inequality is due to the subadditivity of the Toeplitz-modulus. Hence, $D^\rho$ is a metric. $\square$

Because the interest of the present paper is with dilations, further study of ${\mathcal {B}}({\mathcal {H}})^d$ as a $D^\rho$-metric space is not undertaken here. Again, from the point of view of dilations, the metric $D^\rho$ is not entirely satisfying, as the closed unit ball in the $D^\rho$-metric excludes some Toeplitz contractive *d*-tuples, such as the pair $(\sigma ,1_2)$ in Example 4.4.

The following definition is a new generalistion of the numerical radius of an operator *d*-tuple.

### Definition 4.6

If $\textsf{T}=(T_1,\dots ,T_d)\in {\mathcal {B}}({\mathcal {H}})^d$ and $(\alpha ,\xi )\in {\mathbb {R}}_+\times {\mathcal {H}}$, and if ${\mathcal {T}}_{\alpha ,\xi }$ denotes the Toeplitz matrix

4.4 $$\begin{aligned} {\mathcal {T}}_{\alpha ,\xi }= \left[ \begin{array}{ccccc} \alpha &  \langle T_1^*\xi ,\xi \rangle &  \langle T_2^*\xi ,\xi \rangle &  \dots &  \langle T_d^*\xi ,\xi \rangle \\ \langle T_1 \xi ,\xi \rangle &  \alpha &  \langle T_1^*\xi ,\xi \rangle &  \ddots &  \vdots \\ \langle T_2 \xi ,\xi \rangle &  \ddots &  \ddots &  \ddots &  \langle T_2^*\xi ,\xi \rangle \\ \vdots &  \ddots &  \ddots &  \ddots &  \langle T_1^*\xi ,\xi \rangle \\ \langle T_d \xi ,\xi \rangle &  \dots &  \langle T_2 \xi ,\xi \rangle &  \langle T_1 \xi ,\xi \rangle &  \alpha \end{array}\right] , \end{aligned}$$

then the *Toeplitz numerical radius* of $\textsf{T}$ is the nonnegative real number $\omega (\textsf{T})$ defined by

4.5 $$\begin{aligned} \omega (\textsf{T}) =\inf \left\{ \alpha \in {\mathbb {R}}_+\,|\,\mathcal T_{\alpha ,\xi } \hbox { is positive for every unit vector }\xi \in {\mathcal {H}}\right\} . \end{aligned}$$

Observe that the first column of each matrix in (4.4) is determined by $\alpha$ and an element of the spatial numerical range of $\textsf{T}$.

As with the Toeplitz modulus, the Toeplitz numerical radius is a convex function, but is not homogenous for nonpositive or nonreal scalars. However, if $d=1$, then Definition 4.6 yields $\omega (\textsf{T})=w(T_1)$, the classical numerical radius of the operator $T_1$.

The Toeplitz numerical radius is defined spatially. However, as with the classical joint numerical radius, there is a useful alternative description in terms of states on ${\mathcal {B}}({\mathcal {H}})$.

### Proposition 4.7

If $\phi :{\mathcal {B}}({\mathcal {H}})\rightarrow {\mathbb {C}}$ is a state, $\textsf{T}=(T_1,\dots ,T_d)\in {\mathcal {B}}({\mathcal {H}})^d$, and $\alpha \in {\mathbb {R}}_+$, and if ${\mathcal {T}}_{\alpha ,\phi }$ denotes the Toeplitz matrix

4.6 $$\begin{aligned} {\mathcal {T}}_{\alpha ,\phi }= \left[ \begin{array}{ccccc} \alpha &  \phi ( T_1^*) &  \phi (T_2^*) &  \dots &  \phi (T_d^*) \\ \phi ( T_1 ) &  \alpha &  \phi (T_1^*) &  \ddots &  \vdots \\ \phi ( T_2 ) &  \ddots &  \ddots &  \ddots &  \phi (T_2^*) \\ \vdots &  \ddots &  \ddots &  \ddots &  \phi ( T_1^*) \\ \phi ( T_d ) &  \dots &  \phi (T_2) &  \phi (T_1 ) &  \alpha \end{array}\right] , \end{aligned}$$

then

4.7 $$\begin{aligned} \omega (\textsf{T}) =\inf \left\{ \alpha \in {\mathbb {R}}_+\,|\,\mathcal T_{\alpha ,\phi }\; \text {is positive for every state } \phi \; \text { on } {\mathcal {B}}({\mathcal {H}}) \right\} . \end{aligned}$$

### Proof

As every unit vector $\xi \in {\mathcal {H}}$ induces a state $X\mapsto \langle X\xi ,\xi \rangle$ on ${\mathcal {B}}({\mathcal {H}})$, $\omega (\textsf{T})$ is no larger than the quantity on the right side of equation (4.7).

Conversely, assume $\alpha \in {\mathbb {R}}_+$ has the property that ${\mathcal {T}}_{\alpha ,\xi }$ is positive, for every unit vector $\xi$. As every state on ${\mathcal {B}}({\mathcal {H}})$ restricts to a state on the unital $\hbox {C}^*$-algebra $\textrm{C}^*(\textsf{T})$ generated by $T_1,\dots ,T_d$, select a pure state $\phi$ on $\textrm{C}^*(\textsf{T})$. Because $\phi$ is pure and $\textrm{C}^*(\textsf{T})$ is separable, there is a sequence $\{\xi _n\}_{n\in {\mathbb {N}}}$ of unit vectors $\xi _n\in {\mathcal {H}}$ such that $\phi (X)=\displaystyle \lim _{n\rightarrow \infty }\langle X\xi _n,\xi _n\rangle$, for every $X\in \textrm{C}^*(\textsf{T})$ [5, Lemma 2.1]. Hence, the matrices $\mathcal T_{\alpha ,\xi _n}$ converge entrywise to ${\mathcal {T}}_{\alpha ,\phi }$ as $n\rightarrow \infty$, which implies the matrices $\mathcal T_{\alpha ,\xi _n}$ converge in norm to ${\mathcal {T}}_{\alpha ,\phi }$. By hypothesis, ${\mathcal {T}}_{\alpha ,\xi _n}$ is positive for every *n*; hence, ${\mathcal {T}}_{\alpha ,\phi }$ is positive.

Because ${\mathcal {T}}_{\alpha ,\phi }$ is positive for every pure state $\phi$ on $\textrm{C}^*(\textsf{T})$, the same is true for any state $\phi$ on $\textrm{C}^*(\textsf{T})$ that is a convex combination of pure states.

Assume $\phi$ is an arbitrary state on $\textrm{C}^*(\textsf{T})$. By the Krein-Milman Theorem, there is a net $\{\phi _\mu \}_\mu$ of convex combinations of pure states on $\textrm{C}^*(\textsf{T})$ such that $\phi (X)=\displaystyle \lim _\mu \phi _\mu (X)$, for every $X\in \textrm{C}^*(\textsf{T})$. Hence, the net $\{\mathcal T_{\alpha ,\phi _\mu }\}_\mu$ of positive matrices converges pointwise, and hence in norm, to ${\mathcal {T}}_{\alpha ,\phi }$, which proves that ${\mathcal {T}}_{\alpha ,\phi }$ is positive.

Hence, the infimum of $\{ \beta \in {\mathbb {R}}_+\,|\,\mathcal T_{\beta ,\phi }\; \text { is positive for every state } \phi \; \text { on } {\mathcal {B}}({\mathcal {H}}) \}$ is bounded above by $\alpha$, which proves the equality (4.7). $\square$

Although the Toeplitz numerical radius is defined spatially, one advantage in using the definition involving states on ${\mathcal {B}}({\mathcal {H}})$ is that the set of matrices of the form ${\mathcal {T}}_{\alpha ,\phi }$ that are positive, for a fixed $\alpha$ and arbitrary state $\phi$, is convex. In contrast, the spatially defined joint numerical range of a *d*-tuple of operators can fail to be convex, implying that the set of matrices of the form ${\mathcal {T}}_{\alpha ,\xi }$ that are positive, for a fixed $\alpha$ and arbitrary unit vector $\xi$, can fail to be convex.

An inequality [18, §18] arising from the polarisation identity and the characterisation of the operator norm $\Vert T\Vert$ in terms of the bilinear form $(\xi ,\eta )\mapsto \langle T\xi ,\eta \rangle$ on ${\mathcal {H}}\times {\mathcal {H}}$ leads to the following well-known inequalities involving the operator norm and numerical radius:

$$\begin{aligned} \frac{1}{2}\Vert T\Vert \le w(T) \le \Vert T\Vert . \end{aligned}$$

The analogous upper bound for the Toeplitz numerical radius and modulus is straightforward to confirm, as shown by Proposition 4.8 below.

### Proposition 4.8

$$ \omega (\textsf{T})\le \rho (\textsf{T})$$.

### Proof

Suppose that $\alpha \in {\mathbb {R}}_+$ is such that ${\mathcal {T}}_\alpha$ is a positive operator. For any unit vector $\xi \in {\mathcal {H}}$, the positive linear functional $\phi _\xi (X)=\langle X\xi ,\xi \rangle$, for $X\in {\mathcal {B}}({\mathcal {H}})$, is a unital completely positive linear map. Therefore, the matrix $\phi _\xi ^{[d+1]}({\mathcal {T}}_\alpha )$ is positive in ${\mathcal {M}}_{d+1}({\mathbb {C}})$, where $\phi _\xi ^{[d+1]}$ denotes the $(d+1)$-th ampliation of $\phi _\xi$. Because the matrix $\phi _\xi ^{[d+1]}({\mathcal {T}}_\alpha )$ is the matrix given in (4.5), $\alpha$ is an upper bound for $\omega (\mathsf{T)}$. As this is true for any $\alpha \in {\mathbb {R}}_+$ for which $\mathcal T_\alpha$ is a positive operator, $\rho (\textsf{T})$ is also an upper bound for $\omega (\mathsf{T)}$. $\square$

As with the classical numerical radius, the Toeplitz numerical radius coincides with the Toeplitz modulus for *d*-tuples of commuting normal operators. To prove this, it is convenient to introduce a version of spectral radius.

### Definition 4.9

If $\textsf{T}=(T_1,\dots ,T_d)\in {\mathcal {B}}({\mathcal {H}})^d$ is a *d*-tuple of commuting operators and $(\alpha ,\lambda )\in {\mathbb {R}}_+\times \textrm{Sp}_T(\textsf{T})$, and if $\Lambda _{\alpha ,\lambda }$ denotes the Toeplitz matrix

4.8 $$\begin{aligned} \Lambda _{\alpha ,\lambda }= \left[ \begin{array}{ccccc} \alpha &  \overline{\lambda }_1 &  \overline{\lambda }_2 &  \dots &  \overline{\lambda }_d \\ \lambda _1 &  \alpha &  \overline{\lambda }_1 &  \ddots &  \vdots \\ \lambda _2 &  \ddots &  \ddots &  \ddots &  \overline{\lambda }_2 \\ \vdots &  \ddots &  \ddots &  \ddots &  \overline{\lambda }_1 \\ \lambda _d &  \dots &  \lambda _2 &  \lambda _1 &  \alpha \end{array}\right] , \end{aligned}$$

then the *Toeplitz spectral radius* of $\textsf{T}$ is the nonnegative real number $r(\textsf{T})$ defined by

4.9 $$\begin{aligned} r(\textsf{T}) =\inf \left\{ \alpha \in \mathbb R_+\,|\,\Lambda _{\alpha ,\lambda } \hbox { is positive for every }\lambda \in \textrm{Sp}_T(\textsf{T})\right\} . \end{aligned}$$

Further, if *T* is a normal *d*-tuple, then the *Gelfand-Toeplitz spectral radius* of $\textsf{T}$ is the nonnegative real number $r_G(\textsf{T})$ defined by

4.10 $$\begin{aligned} r_G(\textsf{T}) =\inf \left\{ \alpha \in \mathbb R_+\,|\,\Lambda _{\alpha ,\lambda } \hbox { is positive for every }\lambda \in \textrm{Sp}_G(\textsf{T})\right\} . \end{aligned}$$

### Proposition 4.10

If $\textsf{T}$ is a *d*-tuple of commuting operators, then

$$\begin{aligned} r(\textsf{T})\le \omega (\textsf{T})\le \rho (\textsf{T}). \end{aligned}$$

If $\textsf{N}$ is a normal *d*-tuple, then

$$\begin{aligned} r_G(\textsf{N})=r(\textsf{N})=\omega (\textsf{N})=\rho (\textsf{N}). \end{aligned}$$

### Proof

By [28, Corollary 2.3], each $\lambda \in \textrm{Sp}_T(\textsf{T})$ lies in the closure of the spatial numerical range of $\textsf{T}$. Thus, if $\alpha \in \mathbb R_+$ is such that the matrix ${\mathcal {T}}_{\alpha ,\xi }$ is positive for every unit vector $\xi$, then the matrix $\Lambda _{\alpha ,\lambda }$ is positive for every $\lambda \in \textrm{Sp}_T(\textsf{T})$. Hence, $r(\textsf{T})\le \alpha$, which implies $r(\textsf{T})\le \omega (\textsf{T})$.

Suppose now that $\textsf{N}$ is normal. If $\pi :\textrm{C}^*(\textsf{N})\rightarrow C\left( \textrm{Sp}_G(\textsf{N})\right)$ is the unital $*$-isomorphism representing the unital abelian $\hbox {C}^*$-algebra $\textrm{C}^*(\textsf{N})$ as the $\hbox {C}^*$-algebra $C\left( \textrm{Sp}_G(\textsf{N})\right)$ of continuous complex-valued functions on its maximal ideal space $\textrm{Sp}_G(\textsf{N})$, then each $\pi (N_k)$ is the function $\lambda \mapsto \lambda _k$. Furthermore, the ampliation

$$\begin{aligned} \pi ^{[d+1]}:{\mathcal {M}}_{d+1}\left( \textrm{C}^*(\textsf{N})\right) \rightarrow {\mathcal {M}}_{d+1}\left( C(\textrm{Sp}_G(\textsf{N}))\right) , \end{aligned}$$

in which $[X_{ij}]_{i,j}\mapsto [\pi (X_{ij}]_{i,j}$, is also a unital $*$-ismorphism.

Assume $\alpha \in {\mathbb {R}}_+$ is such that $\Lambda _{\alpha ,\lambda }$ is positive for every $\lambda \in \textrm{Sp}_G(\textsf{N})$. Thus, the matrix-valued function $F_\alpha :\textrm{Sp}_G(\textsf{N})\rightarrow {\mathcal {M}}_{d+1}({\mathbb {C}})$ given by $F_\alpha (\lambda )=\Lambda _{\alpha , \lambda }$ is a positive element in the $\hbox {C}^*$-algebra ${\mathcal {M}}_{d+1}\left( C(\textrm{Sp}_G(\textsf{N}))\right)$. Since $F_\alpha =\pi ^{[d+1]}({\mathcal {T}}_{\alpha })$ and because $\pi ^{[d+1]}$ is a $*$-isomorphism, ${\mathcal {T}}_\alpha$ is positive. Hence, $\rho (\textsf{N})\le \alpha$, which implies $\rho (\textsf{N})\le r_G(\textsf{N})$. On the other hand, $r_G(\textsf{N})\le r(\textsf{N})$ by definition, and so $r_G(\textsf{N})\le \rho (\textsf{N})$. $\square$

### Corollary 4.11

If $\textsf{S}$ is a subnormal *d*-tuple, then $r(\textsf{S})=\omega (\textsf{S})=\rho (\textsf{S})$.

### Proof

If $\textsf{N}$ is a minimal normal extension for $\textsf{S}$, then

$$\begin{aligned} \textrm{Sp}_T(\textsf{N})\subseteq \textrm{Sp}_T(\textsf{S})\subseteq \overline{W(\textsf{S})} \subseteq \overline{W(\textsf{N})}, \end{aligned}$$

where the first two inclusions are established in [23] and [28, Corollary 2.3], respectively. Because Proposition 4.10 shows the Gelfand-Toeplitz spectral radius of $\textsf{N}$ coincides with the Toeplitz numerical radius and modulus of $\textsf{N}$, we deduce $r(\textsf{S})=\omega (\textsf{S})=\rho (\textsf{S})$. $\square$

## Embedding $S^1$ into ${\mathbb {C}}^d$ and matrix convexity

### Definition 5.1

Given a Hilbert space ${\mathcal {H}}$, $d\in {\mathbb {N}}$, and $r\in {\mathbb {R}}_+$, let

$$\begin{aligned} \text {Toepl} _{d,{\mathcal {H}}}^\rho (r)=\left\{ \textsf{T}\in {\mathcal {B}}({\mathcal {H}})^d\,|\,\rho (\textsf{T})\le r\right\} \end{aligned}$$

and

$$\begin{aligned} \text {Toepl} _{d,{\mathcal {H}}}^\omega (r)=\left\{ \textsf{T}\in {\mathcal {B}}({\mathcal {H}})^d\,|\,\omega (\textsf{T})\le r\right\} . \end{aligned}$$

If ${\mathcal {H}}={\mathbb {C}}^n$ for some $n\in {\mathbb {N}}$, then $\text {Toepl} _{d,{\mathcal {H}}}^\rho (r)$ and $\text {Toepl} _{d,{\mathcal {H}}}^\omega (r)$ are denoted by $\text {Toepl} _{d,n}^\rho (r)$ and $\text {Toepl} _{d,n}^\omega (r)$.

If ${\mathcal {K}}$ is any Hilbert space, and $X:{\mathcal {K}}\rightarrow {\mathcal {H}}$ is a bounded linear operator, then *X* induces a linear map ${\mathcal {B}}({\mathcal {H}})^d\rightarrow {\mathcal {B}}({\mathcal {K}})^d$, denoted by $\textsf{T}\mapsto X^*\cdot \textsf{T} \cdot X$, and defined by

$$\begin{aligned} X^*\cdot \textsf{T}\cdot X= X^*\cdot (T_1,\dots ,T_d)\cdot X =(X^*T_1X, \dots , X^*T_dX). \end{aligned}$$

### Definition 5.2

An *operator convex combination* of $\textsf{T}_1,\dots ,\textsf{T}_\ell \in {\mathcal {B}}({\mathcal {H}})^d$ is a sum of the form

$$\begin{aligned} \sum _{k=1}^\ell X_k^* \cdot \textsf{T}_k \cdot X_k, \end{aligned}$$

where $X_1,\dots , X_\ell$ are any bounded linear operators ${\mathcal {K}}\rightarrow {\mathcal {H}}$, for any Hilbert space ${\mathcal {K}}$, for which

$$\begin{aligned} \sum _{k=1}^\ell X_k^*X_k=1_{\mathcal {K}}. \end{aligned}$$

A subset of ${\mathcal {B}}({\mathcal {H}})^d$ which is closed under all possible operator convex combinations is said to be *operator convex*.

### Proposition 5.3

For every $d\in {\mathbb {N}}$, Hilbert space ${\mathcal {H}}$, and $r\in \mathbb R_+$, the sets $\text {Toepl} _{d,{\mathcal {H}}}^\rho (r)$ and $\text {Toepl} _{d,{\mathcal {H}}}^\omega (r)$ are operator convex.

### Proof

Because $\rho (r\textsf{T})=r\rho (\textsf{T})$, we may assume without loss of generality that $r=1$. Suppose $\textsf{T}_1,\dots ,\textsf{T}_\ell \in {\mathcal {B}}({\mathcal {H}})^d$ are Toeplitz contractive, ${\mathcal {K}}$ is a Hilbert space, and $X_1,\dots , X_\ell :{\mathcal {K}}\rightarrow {\mathcal {H}}$ are operators satisfying $\displaystyle \sum \nolimits _{k=1}^\ell X_k^*X_k=1_{\mathcal {K}}$. In writing

$$\begin{aligned} \textsf{T}_k=(T_{k1},\dots ,T_{kd}) \hbox { and } \textsf{S} =(S_1, \dots , S_d) = \sum _{k=1}^d X_k^* \cdot \textsf{T}_k \cdot X_k, \end{aligned}$$

we obtain

5.1 $$\begin{aligned} S_j=\sum _{k=1}^\ell X_k^*T_{kj} X_k, \hbox { for each }j=1,\dots , d. \end{aligned}$$

Thus,

$$\begin{aligned} \left[ \begin{array}{ccccc} 1_{\mathcal {H}}&  S_1^* &  S_2^* &  \dots &  S_d^* \\ S_1 &  1_{\mathcal {H}}&  S_1^* &  \ddots &  \vdots \\ S_2 &  \ddots &  \ddots &  \ddots &  S_2^* \\ \vdots &  \ddots &  \ddots &  \ddots &  S_1^* \\ S_d &  \dots &  S_2 &  S_1 &  1_{\mathcal {H}}\end{array}\right] = \sum _{k=1}^\ell {\tilde{X}}_k^* \left[ \begin{array}{ccccc} 1_{\mathcal {H}}&  T_{k1}^* &  T_{k2}^* &  \dots &  T_{kd}^* \\ T_{k1} &  1_{\mathcal {H}}&  T_{k1}^* &  \ddots &  \vdots \\ T_{k2} &  \ddots &  \ddots &  \ddots &  T_{k2}^* \\ \vdots &  \ddots &  \ddots &  \ddots &  T_{k1}^* \\ T_{kd} &  \dots &  T_{k2}&  T_{k1} &  1_{\mathcal {H}}\end{array}\right] {\tilde{X}}_k, \end{aligned}$$

where ${\tilde{X}}_k = \hbox {Diag}(X_k, X_k, \dots X_k)$. As the right side of the equation above is a sum of positive operators, $\textsf{S}$ is a Toeplitz contractive *d*-tuple.

Similarly, to prove $\text {Toepl} _{d,{\mathcal {H}}}^\omega (1)$ is operator convex for $r=1$, select any unit vector $\eta \in {\mathcal {K}}$ and consider, using the assumptions and notation established above, the matrix ${\mathcal {S}}_{1,\eta }$. Let ${\hat{\eta }}_k$ be 0, if $X_k\eta =0$, or $\Vert X_k\eta \Vert ^{-1}(X_k\eta )$ otherwise. By equation (5.1),

$$\begin{aligned} \langle S_j\eta ,\eta \rangle = \sum _{k=1}^\ell \langle T_{kj}X_k\eta , X_k\eta \rangle = \sum _{k=1}^\ell \Vert X_k\eta \Vert ^2\langle T_{kj} {\hat{\eta }}_k, {\hat{\eta }}\rangle , \end{aligned}$$

for each $j=1,\dots ,d$, and so

$$\begin{aligned} {\mathcal {S}}_{1,\eta } = \sum _{k=1}^\ell \Vert X_k\eta \Vert ^2 \mathcal T_{1,{\hat{\eta }}_k}. \end{aligned}$$

Since $\textsf{T}\in \text {Toepl} _{d,{\mathcal {H}}}^\omega (1)$, the matrices ${\mathcal {T}}_{1,{\hat{\eta }}_k}$ are positive, implying the matrix ${\mathcal {S}}_{1,\eta }$ is positive. $\square$

Proposition 5.3 shows that the (graded) set of all Toeplitz contractive operators is a noncommutative convex set in the sense of [20]; likewise, for the set of all *d*-tuples of operators with Toeplitz numerical radius 1 (or any $r>0$). However, for the remainder of this section, we shall require only the notion of a matrix convex set.

### Definition 5.4

A *matrix convex set in* *d* *free dimensions* is a graded set ${\mathbb {K}}$ of the form ${\mathbb {K}}=\displaystyle \bigcup _{n\in \mathbb N} {\mathbb {K}}_n$, where

1. $${\mathbb {K}}_n\subseteq {\mathcal {M}}_n({\mathbb {C}})^d$$, and
2. for all $\ell ,m,n_1,\dots ,n_\ell \in {\mathbb {N}}$, $A_j\in {\mathbb {K}}_{n_j}$, and linear maps $X_j:{\mathbb {C}}^m\rightarrow {\mathbb {C}}^{n_j}$ such that $\displaystyle \sum _{j=1}^\ell X_j^*X_j=1_m$, we have
  $$\begin{aligned} \sum _{j=1}^\ell X_j^*\cdot A_j \cdot X_j\in {\mathbb {K}}_n. \end{aligned}$$

Define $\text {Toepl} _{d}^\rho (r)$ and $\text {Toepl} _{d}^\omega (r)$ to be the graded sets

$$\begin{aligned} \text {Toepl} _{d}^\rho (r)=\displaystyle \bigcup _{n\in \mathbb N}\text {Toepl} _{d,n}^\rho (r)\,\hbox { and }\, \text {Toepl} _{d}^\omega (r)=\displaystyle \bigcup _{n\in \mathbb N}\text {Toepl} _{d,n}^\omega (r), \end{aligned}$$

respectively. Proposition 5.3 shows that each of $\text {Toepl} _{d}^\rho (r)$ and $\text {Toepl} _{d}^\omega (r)$ are matrix convex. Because the cone of positive operators is norm-closed, these matrix convex sets are also compact, which is to say each of the sets at the *n*-th grading is compact in ${\mathcal {M}}_n({\mathbb {C}})^d$. Another class of compact matrix convex sets arise from the matrix ranges (Definition 2.7, defining condition (2.3)) of operator *d*-tuples $\textsf{T}\in {\mathcal {B}}({\mathcal {H}})$.

### Definition 5.5

The *matrix range* of $\textsf{T}\in {\mathcal {B}}({\mathcal {H}})^d$ is the graded set ${\mathbb {W}}(\textsf{T})$ defined by

$$\begin{aligned} {\mathbb {W}}(\textsf{T})= \bigcup _{n\in {\mathbb {N}}} W_n(\textsf{T}). \end{aligned}$$

The matrix convexity of ${\mathbb {W}}(\textsf{T})$ is evident from the fact that linear maps of the form $A\mapsto \displaystyle \sum \nolimits _{j=1}^\ell X_j^* A X_j$ are completely positive, and are unital when $\displaystyle \sum \nolimits _{j=1}^\ell X_j^* X_j$ is the identity matrix.

### Theorem 5.6

The following statements are equivalent for $\textsf{A}=(A_1,\dots ,A_d)\in {\mathcal {M}}_n({\mathbb {C}})^d$;

1. $\textsf{A}\in {\mathbb {W}}(\textsf{W})$, the unitary-power tuple determined by the bilateral shift operator *W*;
2. $\textsf{A}$ is Toeplitz contractive;
3. there exist $\ell \in {\mathbb {N}}$, $\lambda _1,\dots ,\lambda _\ell \in S^1$, and $Q_1,\dots ,Q_\ell \in {\mathcal {M}}_n({\mathbb {C}})_+$ such that
  $$\begin{aligned} \sum _{j=1}^\ell Q_j=1_n\hbox { and } A_k=\sum _{j=1}^\ell \lambda _j^k Q_j, \hbox { for all }k=1,\dots ,d. \end{aligned}$$

### Proof

If $\textsf{A}\in {\mathbb {W}}(\textsf{W})$, then there is a ucp map $\phi :{\mathcal {B}}(\ell ^2({\mathbb {Z}}))\rightarrow {\mathcal {M}}_n({\mathbb {C}})$ such that $\phi (W^k)=A_k$, for all $k=1,\dots , d$. Thus, the matrix $\mathcal A_{1}$ is given by $\phi ^{[d+1]}({\mathcal {W}}_1)$. The positivity of ${\mathcal {W}}_1$ implies, therefore, the positivity of $\mathcal A_{1}$, which is the assertion that the *d*-tuple $\textsf{A}$ is Toeplitz contractive.

Next, assume $\textsf{A}$ is Toeplitz contractive. As the matrix ${\mathcal {A}}_1$ is a $(d+1)\times (d+1)$ matrix with entries that are $n\times n$ matrices $A_k$, ${\mathcal {A}}_1$ can be viewed as a positive element of ${\mathcal {M}}_{d+1}\otimes {\mathcal {M}}_n({\mathbb {C}})$. By the Gurvits separability theorem [13, Corollary 3.3], the positive block-Toeplitz matrix ${\mathcal {A}}_1$ is separable and, moreover, each entry of ${\mathcal {A}}_1$ admits a decomposition of the form $A_k=\displaystyle \sum \nolimits _{j=1}^\ell \lambda _j^k Q_j$, where $\lambda _1,\dots ,\lambda _\ell \in S^1$ and $Q_1,\dots ,Q_\ell \in {\mathcal {M}}_n({\mathbb {C}})_+$ are such that $\displaystyle \sum \nolimits _{j=1}^\ell Q_j=1_n$.

Finally, assume $A_1,\dots ,A_d\in {\mathcal {B}}({\mathcal {H}})$ admit decompositions as indicated in (3): $\displaystyle \sum \nolimits _{j=1}^\ell Q_j=1_n$ and $A_k=\displaystyle \sum \nolimits _{j=1}^\ell \lambda _j^k Q_j$, for some $\lambda _1,\dots ,\lambda _\ell \in S_1$. Because $\text {Sp} (W)=S^1$, there exist characters $\pi _j:\textrm{C}^*(\textsf{W})\rightarrow {\mathbb {C}}$, for $j=1,\dots ,\ell$, such that $\pi _j(W^k)=\lambda _j^k$, for $k=1,\dots ,d$. Therefore, the linear map $\phi :\textrm{C}^*(\textsf{W})\rightarrow {\mathcal {M}}_n({\mathbb {C}})$ defined by $\phi (X)=\displaystyle \sum \nolimits _{j=1}^\ell \pi _j(X)Q_j$, for $X\in \textrm{C}^*(\textsf{W})$, is unital and completely positive, and has the property that $\textsf{A}=\phi (\textsf{W})$. Hence, $\textsf{A}\in {\mathbb {W}}(\textsf{W})$. $\square$

The theory of minimal and maximal matrix convex sets [21] concerns those matrix convex sets ${\mathbb {K}}$ for which ${\mathbb {K}}_1$ is some prescribed compact convex set $\Omega \subseteq {\mathbb {C}}^d$. As demonstrated in [21], among all possibilities, there are a smallest and a largest such matrix convex set, and these are denoted by ${\mathbb {K}}^{\textrm{min}}(\Omega )$ and ${\mathbb {K}}^\textrm{max}(\Omega )$, respectively. These matrix convex sets have rather precise descriptions: a *d*-tuple $\textsf{A}\in {\mathbb {K}}^\textrm{min}(\Omega )$ if and only if there exists a normal dilation $\textsf{N}$ of $\textsf{A}$ such that $\text {Sp} _G(\textsf{N})\subseteq \Omega$, whereas a *d*-tuple $\textsf{B}\in {\mathbb {K}}^\textrm{max}(\Omega )$ if and only if the joint numerical range of $\textsf{B}$ is a subset of $\Omega$.

### Definition 5.7

A *power stretching of* $S^1$ *across* *d* *complex dimensions* is the set ${\mathbb {S}}_d^1\subseteq {\mathbb {C}}^d$ defined by

$$\begin{aligned} {\mathbb {S}}_d^1=\left\{ (\lambda ,\lambda ^2,\dots ,\lambda ^d)\in \mathbb C^d\,|\,\lambda \in S^1\right\} . \end{aligned}$$

Observe that ${\mathbb {S}}_d^1$ is a subgroup of the *d*-torus, isomorphic to the group $S^1$, which is why ${\mathbb {S}}_d^1$ is referred to as a “stretching” of $S^1$. For our purposes, the importance of ${\mathbb {S}}_d^1$ lies in the fact that it coincides with Gelfand joint spectrum of the unitary-power *d*-tuple $\textsf{W}=(W,W^2,\dots , W^d)$, where *W* is the bilateral shift operator on $\ell ^2({\mathbb {Z}})$.

### Theorem 5.8

If $$\textsf{A}, \textsf{B}\in {\mathcal {M}}_n({\mathbb {C}})^d$$, then

1. $\textsf{A}\in {\mathbb {K}}^{\textrm{min}}(\text {conv} \,{\mathbb {S}}_d^1)$ if and only if $\rho (\textsf{A})\le 1$, and
2. $\textsf{B}\in {\mathbb {K}}^{\textrm{max}}(\text {conv} \,{\mathbb {S}}_d^1)$ if and only if $\omega (\textsf{B})\le 1$.

### Proof

To prove (1), assume assume $\textsf{A}\in {\mathbb {K}}^{\textrm{min}}(\text {Conv} \,{\mathbb {S}}_d^1)$. Then, by definition of ${\mathbb {K}}^{\textrm{min}}(\cdot )$, $\textsf{A}$ admits a unitary-power dilation $\textsf{U}$ such that the joint spectrum of $\textsf{U}$ is a subset of ${\mathbb {S}}_d^1$. Thus, $\rho (\textsf{A})\le \rho (\textsf{U})=1$.

Conversely, if $\textsf{A}$ is a *d*-tuple of matrices such that $\rho (\textsf{A})\le 1$, then, by Theorem 3.1, $\textsf{A}$ admits a unitary-power dilation $\textsf{U}$ such that the joint spectrum of $\textsf{U}$ is a subset of ${\mathbb {S}}_d^1$. Hence, $\textsf{A}\in {\mathbb {K}}^{\textrm{min}}(\text {Conv} \,{\mathbb {S}}_d^1)$.

To prove (2), assume $\textsf{B}\in {\mathbb {K}}^{\textrm{max}}(\text {Conv} \,{\mathbb {S}}_d^1)$, select a unit vector $\xi$, and consider the matrix ${\mathcal {B}}_{1,\xi }\in {\mathcal {M}}_{d+1}({\mathbb {C}})$. By hypothesis, $W^1(\textsf{B})\in \text {Conv} \,{\mathbb {S}}_d^1$; thus, there exist $\ell \in {\mathbb {N}}$, $t_1,\dots ,\hbox {tr }_\ell \in {\mathbb {R}}_+$, and $\lambda _1,\dots ,\lambda _\ell \in {\mathbb {S}}^1$ such that $\displaystyle \sum \nolimits _{j=1}^\ell t_j=1$ and $\langle B_k\xi ,\xi \rangle =\displaystyle \sum \nolimits _{j=1}^\ell t_j\lambda _j^k$, for each $k=1,\dots ,d$. Therefore, $\mathcal B_{1,\xi }=\displaystyle \sum \nolimits _{j=1}^\ell t_j T_{d+1}(\lambda _j)$, which is a convex combination of positive matrices $T_{d+1}(\lambda _j)$ (as defined in Definition 2.8). Hence, ${\mathcal {B}}_{1,\xi }$ is positive. Since the choice of $\xi$ is arbitrary, this establishes $\omega (\textsf{B})\le 1$.

Conversely, if $\textsf{B}$ is a *d*-tuple of matrices such that $\omega (\textsf{B})\le 1$, then, by Theorem 2.9, for any unit vector $\xi$, the positive matrix ${\mathcal {B}}_{1,\xi }$ is a convex combination ${\mathcal {B}}_{1,\xi }=\displaystyle \sum \nolimits _{j=1}^\ell t_j T_{d+1}(\lambda _j)$ of matrices of the form $T_{d+1}(\lambda _j)$, for some $\lambda _1,\dots ,\lambda _\ell \in {\mathbb {S}}^1$. Hence, $\langle B_k,\xi ,\xi \rangle =\displaystyle \sum \nolimits _{j=1}^\ell t_j\lambda _j^k$, for each $k=1,\dots ,d$, which implies $W^1(\textsf{B})\subseteq \text {Conv} \,{\mathbb {S}}_d^1$. That is, $\textsf{B}\in \mathbb K^{\textrm{max}}(\text {Conv} \,{\mathbb {S}}_d^1)$. $\square$

Because convexity theory is generally undertaken in the setting of real vectors, we shall need to pass to real and imaginary parts for both spaces and operators. In considering ${\mathbb {C}}^d$ as $\mathbb R^d+i{\mathbb {R}}^d\cong {\mathbb {R}}^{2d}$, ${\mathbb {C}}^d$ is a real vector space of real dimension 2*d*, and convex subsets $\Omega \subseteq {\mathbb {C}}^d$ are also convex when considered as subsets of ${\mathbb {R}}^{2d}$. Importantly, if $\mathbb B_{\ell ^2(n)}^{{\mathbb {C}}}$ and ${\mathbb {B}}_{\ell ^2(n)}^{{\mathbb {R}}}$ denote the closed unit balls of ${\mathbb {C}}^n$ and ${\mathbb {R}}^n$, respectively, with respect to the Euclidean $\ell ^2$ norm, then

$$\begin{aligned} {\mathbb {B}}_{\ell ^2(d)}^{{\mathbb {C}}}={\mathbb {B}}_{\ell ^2(2d)}^{\mathbb R}, \end{aligned}$$

in considering ${\mathbb {B}}_{\ell ^2(d)}^{{\mathbb {C}}}$ as a subset of ${\mathbb {R}}^{2d}$.

### Proposition 5.9

$$\frac{1}{\sqrt{d}}{\mathbb {B}}_{\ell ^2(d)}^{{\mathbb {C}}} \subseteq \text {Conv} \,{\mathbb {S}}_d^1 \subseteq \sqrt{d}\,{\mathbb {B}}_{\ell ^2(d)}^{{\mathbb {C}}}$$.

### Proof

The inclusion $\text {Conv} \,{\mathbb {S}}_d^1 \subseteq \sqrt{d}\,{\mathbb {B}}_{\ell ^2(d)}^{{\mathbb {C}}}$ follows from the fact that every element of ${\mathbb {S}}_d^1$ has norm $\sqrt{d}$.

Conversely, select a vector $\xi \in {\mathbb {C}}^d$ of norm $\Vert \xi \Vert \le \frac{1}{\sqrt{d}}$, and consider the Toeplitz matrix $\Lambda _{1,\xi }$. To show that $\xi \in \text {Conv} \,{\mathbb {S}}_d^1$, it is sufficient to prove that $\Lambda _{1,\xi }$ is positive. Thus, by the Geršgorin Circle Theorem, it is sufficient to show the sum of the moduli of the off-diagonal entries in each row (or column) of $\Lambda _{1,\xi }$ is no larger than 1. Because of the Toeplitz structure of $\Lambda _{1,\xi }$, it sufficient to show this for the first column. By the Cauchy-Schwarz inequality,

$$\begin{aligned} \sum _{j=1}^{d} |\xi _j| \le \sqrt{d} \left( \sum _{j=1}^{d} |\xi _j|^2 \right) ^{1/2} = \sqrt{d}\, \Vert \xi \Vert \le 1. \end{aligned}$$

Thus, $\Lambda _{1,\xi }$ is positive, and so $\xi \in \text {Conv} \,{\mathbb {S}}_d^1$. $\square$

### Corollary 5.10

The convex hull of ${\mathbb {S}}_d^1$ is a convex body in ${\mathbb {C}}^d$ containing 0 in its interior.

The importance of Corollary lies in the fact that the results of [21, §3.2] can be applied to $\text {Conv} \,{\mathbb {S}}_d^1$. To this end, the following Banach-Mazur-type distance, applicable to nonsymmetric convex bodies, was used to great value in [21]. Namely, if *K* and *L* are two compact convex subsets of ${\mathbb {R}}^{2d}$ such that $K \cap L\supseteq \{0\}$, then

$$\begin{aligned} \delta (K,L)=\inf \left\{ C>0\,|\,\hbox {there exists }R\in \text {GL} _{2d}({\mathbb {R}})\hbox { such that } K\subseteq R(L) \subseteq C\cdot K\right\} \end{aligned}$$

is finite.

In the case where $K={\mathbb {B}}_{\ell ^2(d)}^{{\mathbb {C}}}$ and $L=\text {Conv} \,{\mathbb {S}}_d^1$, Proposition 5.9 shows that

$$\begin{aligned} \delta \left( {\mathbb {B}}_{\ell ^2(d)}^{\mathbb C},\text {Conv} {\mathbb {S}}_d^1\right) \le d, \end{aligned}$$

where in the set for which $\delta$ is the infimum, one choice of *R* and *C* is given by $R=\sqrt{d}\cdot 1_{2d}$ and $C=\sqrt{d}\,\sqrt{d}=d$.

### Theorem 5.11

([21, §5]) If $\Omega$ is a compact convex body in ${\mathbb {C}}^d$ with 0 in its interior, then there exists a positive constant $\theta (\Omega )$ such that

1. $${\mathbb {K}}^{\textrm{max}}(\Omega ) \subseteq \theta (\Omega ) \cdot {\mathbb {K}}^{\textrm{min}}(\Omega )$$,
2. $$\theta (\Omega )=\min \left\{ C>0\,|\,{\mathbb {K}}^{\textrm{max}}(\Omega ) \subseteq C \cdot {\mathbb {K}}^{\textrm{min}}(\Omega )\right\} $$, and
3. $$2d \le \delta \left( {\mathbb {B}}_{\ell ^2(d)}^{{\mathbb {C}}},\Omega \right) \,\theta (\Omega )$$.

The final main result of this paper is an analogue, for the Toeplitz modulus and numerical radius, of the familar inequality $\Vert T\Vert \le 2w(T)$ in operator theory. However, the exact value of the constant $c_d$ is not known for $d\ge 2$.

### Theorem 5.12

For every $d\in {\mathbb {N}}$, there exists a constant $c_d\ge 2$ such that

$$\begin{aligned} \rho (\textsf{T})\le c_d\cdot \omega (\textsf{T}), \end{aligned}$$

for every operator *d*-tuple $T\in {\mathcal {B}}({\mathcal {H}})^d$.

### Proof

The set $\text {Conv} \,{\mathbb {S}}_d^1$ is a compact, convex body with 0 in its interior, by Proposition 5.9. Therefore, Theorem 5.11 applies to $\text {Conv} \,{\mathbb {S}}_d^1$, leading to the existence of a positive constant $c_d=\theta (\text {Conv} \,{\mathbb {S}}_d^1)$ with the properties

$$\begin{aligned} {\mathbb {K}}^{\textrm{max}}\left( \text {Conv} \,{\mathbb {S}}_d^1\right) \subseteq c_d \cdot {\mathbb {K}}^\textrm{min}\left( \text {Conv} \,{\mathbb {S}}_d^1\right) \end{aligned}$$

and

$$\begin{aligned} 2d \le \delta \left( {\mathbb {B}}_{\ell ^2(d)}^{\mathbb C}\,\text {Conv} \,{\mathbb {S}}_d^1\right) \,c_d. \end{aligned}$$

Because $\delta \left( {\mathbb {B}}_{\ell ^2(d)}^{\mathbb C}\,\text {Conv} \,{\mathbb {S}}_d^1\right) \le d$, we obtain from the inequality above that $c_d\ge 2$.

Suppose now that $\textsf{T}\in {\mathcal {B}}({\mathcal {H}})$ and let $\alpha =\omega (\textsf{T})$. Thus, $\tilde{\textsf{T}}=\frac{1}{\alpha }\textsf{T}$ has Toeplitz numerical radius $\omega (\tilde{\textsf{T}})=1$. By Theorem 5.8, $\tilde{\textsf{T}}\in {\mathbb {K}}^\textrm{max}\left( \text {Conv} \,{\mathbb {S}}_d^1\right)$. Therefore,

$$\begin{aligned} \tilde{\textsf{T}}\in \cdot {\mathbb {K}}^\textrm{min}\left( \text {Conv} \,{\mathbb {S}}_d^1\right) , \end{aligned}$$

which implies $\rho (c_d^{-1}\tilde{\textsf{T}})\le 1 = \omega (\tilde{T})$ and, hence, $\rho (\textsf{T})\le c_d\cdot \omega (\textsf{T})$. $\square$

## Discussion

The material presented here is inspired by the dilation theorem of Halmos and the theorems of Ando and Gurvits on truncated moments and separable positive block Toeplitz matrices. As explained earlier, the underlying geometry for Halmos’ dilation theorem is the geometry of $\text {Conv} \,S^1$, whereas the geometry inherent to the Ando-Gurvits theorems relies on the stretched version $\mathbb S_d^1$ of $S^1$ and its convex hull. Some earlier works, such as [4, 14], established a dilation theory for operator *d*-tuples in which the normal dilations have joint spectrum within a simplex. Works such as [9, 21] advanced the theory of normal dilations substantially, showing that simplex-structures could be replaced by more general regions. Likewise, in the present paper, the geometry of $\text {Conv} \,{\mathbb {S}}_d^1$ is not that of a simplex, but it is a subset of ${\mathbb {C}}^d$ in which the joint spectrum of unitary power dilations of Toeplitz-contractive *d*-tuples can be found.

It is of interest to determine good estimates, or the exact value, for the constant $c_d$ in Theorem 5.12. The lack of symmetry in $\text {Conv} \,{\mathbb {S}}_d^1$ is an obstacle in drawing upon known estimates for the Banach-Mazur distance between $\mathbb B_{\ell ^2(d)}^{\mathbb {C}}$ and other (usually symmetric) convex bodies.

## Data Availability

No data was available for this work.
